# Impact of home blood pressure variability on cardiovascular outcome in patients with arterial stiffness: Results of the J‐HOP study

**DOI:** 10.1111/jch.14327

**Published:** 2021-07-20

**Authors:** Yusuke Ishiyama, Satoshi Hoshide, Hiroshi Kanegae, Kazuomi Kario

**Affiliations:** ^1^ Division of Cardiovascular Medicine Department of Medicine Jichi Medical University Tochigi Japan; ^2^ Genki Plaza Medical Center for Health Care Tokyo Japan

**Keywords:** arterial stiffness, blood pressure variability, brachial‐ankle pulse wave velocity, cardiovascular disease, home blood pressure

## Abstract

This study sought to investigate whether the relation between increased blood pressure (BP) variability and increased arterial stiffness confers a risk for cardiovascular disease (CVD) events. We analyzed 2648 patients from a practitioner‐based population (mean ± SD age 64.9 ± 11.4 years: 75.8% taking antihypertensive medication) with at least one cardiovascular risk factor who underwent home BP monitoring in the Japan Morning Surge‐Home Blood Pressure Study. The standard deviation (SD_SBP_), coefficient of variation (CV_SBP_), and average real variability (ARV_SBP_) were assessed as indexes of day‐by‐day home systolic BP (SBP) variability. The authors assessed arterial stiffness by brachial‐ankle pulse wave velocity (baPWV) and divided patients into lower (< 1800 cm/s, n = 1837) and higher (≥1800 cm/s, n = 811) baPWV groups. During a mean follow‐up of 4.4 years, 95 cardiovascular events occurred (8.1 per 1000 person‐years). In Cox proportional hazard models adjusted for traditional cardiovascular risk factors including average home SBP, the highest quartiles of SD_SBP_ (hazard ratio [HR], 2.30; 95% confidence interval [CI], 1.23‐4.32), CV_SBP_ (HR, 2.89; 95%CI, 1.59‐5.26) and ARV_SBP_ (HR, 2.55; 95%CI, 1.37‐4.75) were predictive of CVD events compared to the other quartiles in the higher baPWV group. Moreover, 1SD increases in SD_SBP_ (HR, 1.44; 95%CI, 1.13‐1.82), CV_SBP_ (HR, 1.49; 95%CI, 1.16‐1.90) and ARV_SBP_ (HR, 1.37; 95%CI, 1.09‐1.73) were also predictive of CVD events. These associations remained even after N‐terminal pro‐brain natriuretic peptide was added to the models. However, these associations were not observed in the lower baPWV group. We conclude that arterial stiffness contributes to the association between home BP variability and CVD incidence.

## 1 INTRODUCTION

A majority of studies on home blood pressure (BP) readings have reported that BP readings measured at home are associated with an increased risk of cardiovascular disease (CVD) events independent of office BP.^1^, ^2^, ^3^, ^4^, ^5^, ^6^ Therefore, recent guidelines recommend the diagnosis and management of hypertension using home BP monitoring.^7^, ^8^, ^9^ In addition, BP variability assessed by home BP, which has been recognized as day‐by‐day home BP variability, has also been reported to be associated with organ damage and CVD events independent of or beyond mean home BP levels.^10^, ^11^, ^12^, ^13^, ^14^

The relation between BP variability and CVD events has not been fully elucidated, but arterial stiffness has been studied as a potentially potent factor involved in this relation. Increased BP variability has been reported to be associated with greater arterial stiffness, and vice versa.^15^, ^16^, ^17^ As a result, the vicious cycle of increased BP variability and increased arterial stiffness may lead to a risk for CVD events. However, despite the potential importance of such a cycle to the incidence of CVD events, no studies have been performed to verify this proposition.

Using data from the Japan Morning Surge‐Home Blood Pressure (J‐HOP) Study, which enrolled patients with a history of and/or risk factors for CVD and measured their home BP using a validated device in the morning and evening over a 14‐day period, we investigated whether the association between home BP variability and a risk of CVD differs according to increased brachial‐ankle pulse wave velocity (baPWV). The baPWV has been noninvasively measured and used as an index of arterial stiffness in clinical settings and is related to the incidence of CVD events.^18^, ^19^, ^20^ We hypothesized that the day‐by‐day home BP variability may be more useful to predict CVD events in patients with increased baPWV than those without.

## 2 METHODS

### 2.1 Study population

We used baseline data from the Japan Morning Surge‐Home Blood Pressure (J‐HOP) Study. The detailed information of the J‐HOP study has already been published^13^ and is presented in the Supplementary Materials of the present study. Briefly, the J‐HOP study was a prospective observational study conducted to investigate the ability of home BP measurement to predict CVD events in 4310 Japanese ambulatory patients who had a history of and/or risk factors for CVD and were enrolled between January 2005 and May 2012. All participants provided written informed consent for their data to be used, and the Institutional Review Board of Jichi Medical School approved the present study.

Of the 4310 patients enrolled in the J‐HOP study, we excluded 1662 patients who lacked data regarding follow up (n = 32), home BP variability (n = 47) or baPWV (n = 1583). A final total of 2648 patients were included in the present analysis. Diabetes mellitus was defined as fasting glucose ≥126 mg/dL and/or a casual glucose ≥200 mg/dL or treatment for diabetes. Dyslipidemia was defined as total cholesterol ≥240 mg/dL or treatment for dyslipidemia. Chronic kidney disease (CKD) was defined as the presence of proteinuria or an estimated glomerular filtration rate (eGFR) < 60 mL/min/1.73 m^2^. A pre‐existing CVD was defined as diagnosed angina pectoris, myocardial infarction, or stroke.

### 2.2 Office and home BP measurements

Each patient underwent three office BP readings taken at 15‐s intervals on two different clinic visits, measured with an upper arm cuff and an oscillometric BP device (HEM‐5001; Omron Healthcare, Kyoto, Japan). The average of the office BP measurements was used as the patient's office BP level. The self‐measured home BP measurements were taken according to the Japanese guideline.^21^ Each patient measured home BP three times on one occasion at 15‐s intervals in a seated position in both the morning (within 1 h of waking and before taking antihypertensive medication) and evening (before going to bed) for 14 consecutive days using a validated upper arm cuff oscillometric device (HEM‐5001; Omron Healthcare, Kyoto, Japan). Home BP values measured on the first day were excluded, and then we used the averages of the remaining home BP readings to calculate the home mean BP levels. All home BP data were automatically stored in the memory of the BP device and were downloaded to a computer by a physician or nurse at the time of the patient's clinic visits.

We calculated and used the standard deviation (SD_SBP_), the coefficient of variation (CV_SBP_), and the average real variability (ARV_SBP_) for systolic BP (SBP) of the home BP (average of morning and evening SBPs) as the day‐by‐day home BP variability measurement for each individual. These indexes of home BP variability have been used in several other studies.^11^, ^12^, ^22^

### 2.3 Blood samples and baPWV

Blood samples were collected from each patient in the morning in a fasting state at study enrollment. N‐terminal pro‐B‐type natriuretic peptide (NT‐proBNP) was measured with an automated Cobas analyzer using an electrochemiluminescent immunoassay (Roche Diagnostics, Tokyo). The baPWV was measured by using the volume‐plethysmographic method (form PWV/ankle brachial index; Omron Healthcare).^20^ The baPWV was measured by trained investigators in a quiet and temperature‐controlled laboratory after the patient rested for 5 min in the supine position.^23^

### 2.4 Outcome ascertainment

Vital status was ascertained through May 2018, with an average follow‐up period of 4.4 years (11690 person‐years). The primary outcome of this study was a composite of incident CVD events that included fatal or nonfatal coronary heart disease (hospitalization for myocardial infarction and angina pectoris requiring cardiac revascularization) and fatal or non‐fatal stroke.

### 2.5 Statistical analyses

Data are expressed as the mean±SD, the median and interquartile range (IQR), or a percentage. Continuous variables were compared using Student's *t*‐test, and categorical variables were compared using the chi‐squared test between groups. We used the average of the right and left baPWV values for this analysis, and we divided the patients by baPWV into lower baPWV and higher baPWV groups with a cut‐off level of 1800 cm/s according to previous studies.^24^, ^25^, ^26^ After dividing baPWV into lower and higher groups, the Kaplan‐Meier curve of CVD event‐free survival for quartiles of SD_SBP_, CV_SBP_, and ARV_SBP_ in the lower and higher baPWV groups was calculated. Using Cox proportional hazards models, the hazard ratios (HRs) and 95% confidence intervals (CIs) of CVD events associated with the quartiles of each home BP variability index compared to the first quartile as a reference and each home BP variability index as a continuous variable were calculated. Covariates included traditional risk factors such as age, sex, body mass index, diabetes mellitus, total cholesterol, high‐density lipoprotein cholesterol, current smoking, alcohol, pre‐existing CVD, antihypertensive drug use, statin use, and office SBP (Model 1). Model 2 included log‐transformed NT‐proBNP to adjusted factors in model 1. In the secondary analysis, after dividing each home BP variability index into quartiles, we divided baPWV into lower and higher groups and performed Cox proportional hazards model analysis for the association between quartiles of each home BP variability index compared to the first quartile as a reference and each home BP variability index as a continuous variable.

To determine the association between home BP variability and CVD events, we quantified the indirect associations acting through higher baPWV as a mediating variable and direct associations not mediated by home BP variability. We used the Cox proportional hazards model to estimate the direct effect (DE) and the indirect effect (IE) of home BP variability on CVD events. Two models were estimated: (1) a multivariate logistic regression model for higher baPWV (mediator) conditional on home BP variability (exposure) and all study confounders, and (2) a multivariate Cox model for the CVD event (outcome) conditional on home BP variability, higher baPWV, and all study confounders. The DE represented the effect of home BP variability on CVD events that was independent of higher baPWV. The IE represented the proportion of home BP variability that could be explained by its association with changes in higher baPWV. To quantify the magnitude of mediation, we estimated the proportion of the association mediated by higher baPWV (DE*[IE – 1]/[DE*IE – 1]).

All statistical analysis was performed with SPSS ver. 24.0 (IBM, Armonk, NY). *P*‐values < .05 were considered significant for all tests.

## 3 RESULTS

The patients’ clinical characteristics according to the groups which were included or not are summarized in Supple. Table 1. Home BPs of the included population were higher than those of the excluded population, but other characteristics were similar in both populations. The patients' demographic and clinical characteristics according to the groups with lower (< 1800 cm/s) and higher (≥ 1800 cm/s) baPWV values are summarized in Table 1. The patients with higher baPWV values accounted for 30.6% of the study population. Compared to the lower baPWV group, the higher baPWV group had higher age, prevalence of diabetes mellitus and pre‐existing CVD, NT‐proBNP, office SBP and pulse rate (PR), and home SBP and PR, and had lower BMI and total cholesterol. The higher baPWV group had significantly higher indexes of day‐by‐day home BP variability compared to the lower baPWV group.

**TABLE 1:** Baseline clinical characteristics of the patients, based on baPWV values

	baPWV	
Descriptive variable	<1800 cm/s (n = 1837)	≥1800 cm/s (n = 811)	*P* values
Age, y	61.7 ± 10.9	72.3 ± 8.6	<.001
Men, %	45.6	42.8	.185
Body mass index, kg/m^2^	24.8 ± 3.7	23.6 ± 3.1	<.001
Current smoker, %	12.3	9.3	.022
Daily drinker, %	23.4	21.1	.188
Antihypertensive medication, %	75.5	76.7	.490
Diabetes mellitus, %	22.7	27.7	.005
Statin use, %	24.8	23.3	.401
Pre‐existing CVD, %	13.0	16.0	.035
Total cholesterol, mg/dL	201.2 ± 32.2	196.3 ± 33.1	<.001
High‐density lipoprotein, mg/dL	56.9 ± 14.6	56.2 ± 14.7	.263
NT‐proBNP, pg/mL	44.8 (22.5‐87.6)	79.7 (42.9‐165.9)	<.001
baPWV, cm/s	1492 ± 172	2095 ± 286	<.001

BP and PR parameters			
Office SBP, mmHg	138.1 ± 14.8	149.3 ± 17.4	<.001
Office DBP, mmHg	81.7 ± 10.7	80.5 ± 11.3	.013
Office PR, bpm	70.5 ± 10.8	73.0 ± 11.1	<.001
Morning home SBP, mmHg	136.6 ± 14.3	147.1 ± 16.8	<.001
Morning home DBP, mmHg	80.1 ± 10.1	78.8 ± 10.8	.002
Morning PR, bpm	64.9 ± 8.8	66.3 ± 9.2	<.001
Evening home SBP, mmHg	128.0 ± 13.9	136.6 ± 16.2	<.001
Evening home DBP, mmHg	73.2 ± 9.7	72.0 ± 10.4	.005
Evening PR, bpm	69.4 ± 9.3	70.6 ± 9.7	.002
Mean morning & evening SBP, mmHg	132.2 ± 13.1	141.8 ± 15.3	<.001
Mean morning & evening DBP, mmHg	76.6 ± 9.4	75.4 ± 10.2	.002
Mean morning & evening PR, bpm	67.1 ± 8.5	68.4 ± 8.9	<.001

BP variability parameters			
SD of morning SBP, mmHg	7.9 ± 3.2	9.2 ± 3.6	<.001
SD of evening SBP, mmHg	9.3 ± 3.7	10.6 ± 4.1	<.001
SD of morning & evening SBP, mmHg	6.5 ± 2.5	7.4 ± 2.8	<0.001
CV of morning SBP, %	5.8 ± 2.1	6.3 ± 2.3	<.001
CV of evening SBP, %	7.3 ± 2.7	7.8 ± 2.8	<.001
CV of morning & evening SBP, %	4.9 ± 1.8	5.2 ± 1.9	<.001
ARV of morning SBP, mmHg	8.2 ± 3.6	9.6 ± 4.0	<.001
ARV of evening SBP, mmHg	10.3 ± 4.4	11.5 ± 4.8	<.001
ARV of morning & evening SBP, mmHg	6.7 ± 2.9	7.6 ± 3.2	<.001

Data are mean±SD, median (IQR), or percentage. ARV indicates average real variability; baPWV, brachial‐ankle pulse wave velocity; CV: coefficient of variation; CVD, cardiovascular disease; DBP, diastolic blood pressure; IQR, interquartile range; NT‐proBNP, N‐terminal pro‐B‐type natriuretic peptide; SBP, systolic blood pressure; SD, standard deviation; PR, pulse rate.

**FIGURE 1:**
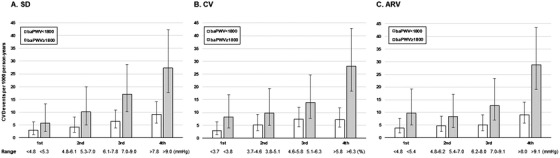
CVD events per 1000 person‐years of (A) SD (B) CV (C) ARV of mean home SBP. *White bars*: The baPWV < 1800 cm/s group. *Gray bars*: The baPWV ≥1800 cm/s group. The error bars show the 95% confidence intervals. Abbreviations: ARV, average real variability; baPWV, brachial‐ankle pulse wave velocity; CV, coefficient of variation; SBP, systolic blood pressure; SD, standard deviation

**FIGURE 2:**
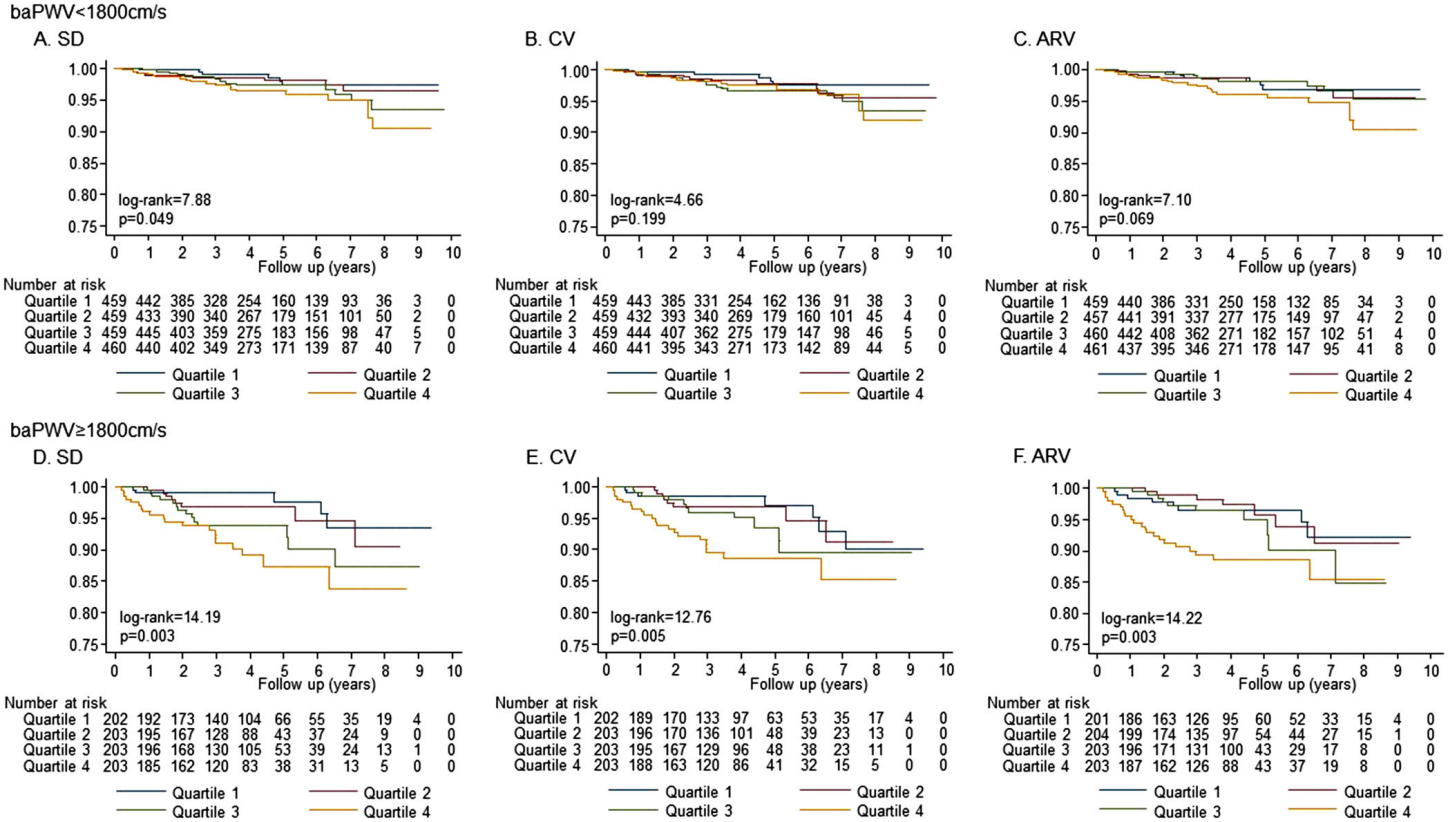
The Kaplan‐Meier curve of the quartiles of blood pressure variability in the groups with lower or higher baPWV value. Abbreviations: ARV, average real variability; baPWV, brachial‐ankle pulse wave velocity; CV, coefficient of variation; SBP, systolic blood pressure; SD, standard deviation

CVD events occurred in 48 patients (5.7 per 1000 person‐years) of the lower baPWV group and 47 patients (14.6 per 1000 person‐years) of the higher baPWV group. Figure 1 shows the incidence rate of CVD events in the cohort separately for the divisions into quartiles of SD, CV, and ARV. Although a stepwise increase in observed CVD events was seen with increasing quartiles in both the groups with lower and higher baPWV, the trend in the group with higher baPWV was significant (all *P* for trend < .01), but the trend in the group with lower baPWV was not (the *P* for trend were .038, .149, and .111 in SD, CV and ARV, respectively). We then performed Kaplan‐Meier survival analysis across quartiles of the indexes of day‐by‐day home BP variability in the lower and higher baPWV groups. The results of the log‐rank test indicated that the indexes of day‐by‐day home BP variability were significantly associated with CVD incidence in the group with higher baPWV (Figure 2). In the Cox proportional hazards model adjusted for other covariates that included average home SBP, the indexes of day‐by‐day home BP variability were incrementally associated with CVD incidence from the lowest quartile to the highest quartile in the group with higher baPWV, whereas this association was not found in the group with lower baPWV (Table 2). Even after adding adjustment for log‐transformed NT‐proBNP, these associations remained. In the secondary analysis, after we divided patients into the quartiles of SD_SBP_, CV_SBP_, and ARV_SBP_, we divided them into groups of lower and higher baPWV, and then performed a similar analysis. The results of CVD incidence (Supple. Figure 1) and the risk of day‐by‐day home BP variability in Cox proportional hazard models (Supple. Table 2) for CVD events were similar.

**TABLE 2:** Hazard ratio of the quartile of BP variability for cardiovascular events in the population with lower or higher baPWV

PWV < 1800 cm/s					PWV ≥ 1800 cm/s			
Quartiles	range	event/number	HR (95%CI)	*P*	range	event/number	HR (95%CI)	*P*
SD of SBP, mmHg								
Quartile 1	<4.8	6/460	1 [Reference]		<5.3	5/202	1 [Reference]	
Quartile 2	4.8‐6.1	9/459	1.17 (0.41–3.32)	.772	5.3–7.0	8/203	1.93 (0.62–5.96)	.254
Quartile 3	6.1‐7.8	14/459	1.40 (0.52–3.75)	.501	7.0–9.0	14/203	3.61 (1.28–10.18)	.015
Quartile 4	>7.8	19/459	1.50 (0.56–4.04)	.423	>9.0	20/203	4.84 (1.74–13.47)	.003
CV of SBP, %								
Quartile 1	<3.7	6/460	1 [Reference]		<3.8	7/202	1 [Reference]	
Quartile 2	3.7‐4.6	11/459	1.30 (0.47–3.56)	.613	3.8‐5.1	8/203	1.37 (0.49–3.84)	.548
Quartile 3	4.6‐5.8	16/459	1.61 (0.61–4.20)	.335	5.1‐6.3	11/203	1.96 (0.74–5.14)	.173
Quartile 4	>5.8	15/459	1.41 (0.53–3.73)	.487	>6.3	21/203	4.05 (1.68–9.76)	.002
ARV of SBP, mmHg								
Quartile 1	<4.8	8/460	1 [Reference]		<5.4	8/201	1 [Reference]	
Quartile 2	4.8‐6.2	10/460	0.77 (0.30–1.98)	.581	5.4‐7.0	7/204	0.82 (0.29–2.28)	.698
Quartile 3	6.2‐8.0	11/458	0.82 (0.32–2.09)	.684	7.0‐9.1	10/203	1.37 (0.53–3.57)	.515
Quartile 4	>8.0	19/459	1.06 (0.43–2.58)	.906	>9.1	22/203	2.71 (1.14–6.43)	.023

We divided into quartiles by SBP variability after dividing into two groups by baPWV. Adjusted factors for age, sex, body mass index, diabetes, total‐cholesterol, high‐density lipoprotein cholesterol, smoking, alcohol, pre‐existing cardiovascular disease, the use of antihypertensive drug and statin, office SBP, and average home SBP. ARV indicates average real variability; baPWV, brachial‐ankle pulse wave velocity; CI, confidence interval; CV, coefficient of variation; HR, hazard ratio; SBP, systolic blood pressure; SD, standard deviation.

In addition, the highest quartiles of SD_SBP_, CV_SBP_, and ARV_SBP_ were significantly associated with CVD incidence in the group with higher baPWV (all *P* < .05), but not in the group with lower baPWV (Table 3). There was an interaction between CV_SBP_ and CVD events according to the lower and higher baPWV group (*P* = .047). When we performed a Cox proportional hazards model for each day‐by‐day home BP variability per 1SD as a continuous variable for CVD events in the group with lower and higher baPWV adjusted for covariates including average home SBP (model 1) and plus log‐transformed NT‐proBNP (model 2) (Table 3), the SD_SBP_, CV_SBP_, and ARV_SBP_ per 1SD were significantly associated with CVD incidence in the group with higher baPWV (all *P* < .05), but not in the group with lower baPWV.

**TABLE 3:** Hazard ratio of the highest quartile of BP variability and BP variability per 1SD for cardiovascular events in the population with lower and higher baPWV

	baPWV < 1800 cm/s		baPWV ≥ 1800 cm/s	
Cardiovascular events/number of patients	48/1837		47/811		P for interaction
	HR (95% CI)	*P*	HR (95% CI)	*P*	
Category of BP variability					
Model 1					
Highest quartile of SD of SBP	1.20 (0.64‐2.27)	.569	2.30 (1.23‐4.32)	.009	0.256
Highest quartile of CV of SBP	1.04 (0.56‐1.94)	.904	2.89 (1.59‐5.26)	.001	0.047
Highest quartile of ARV of SBP	1.26 (0.68‐2.36)	.465	2.55 (1.37‐4.75)	.003	0.263
Model 2					
Highest quartile of SD of SBP	1.09 (0.54‐2.21)	.819	2.05 (1.03‐4.07)	.040	0.310
Highest quartile of CV of SBP	0.94 (0.47‐1.88)	.852	2.30 (1.19‐4.42)	.013	0.149
Highest quartile of ARV of SBP	1.14 (0.57‐2.27)	.712	2.31 (1.17‐4.57)	.016	0.353
Continuous BP variability per 1SD					
Model 1					
SD of SBP	1.20 (0.90‐1.60)	.222	1.44 (1.13‐1.82)	.003	0.759
CV of SBP	1.17 (0.89‐1.55)	.259	1.49 (1.16‐1.90)	.002	0.487
ARV of SBP	1.11 (0.88‐1.41)	.382	1.37 (1.09‐1.73)	.007	0.580
Model 2					
SD of SBP	1.08 (0.77‐1.51)	.655	1.37 (1.05‐1.80)	.020	0.728
CV of SBP	1.07 (0.78‐1.48)	.665	1.42 (1.07‐1.87)	.015	0.554
ARV of SBP	1.02 (0.76‐1.35)	.921	1.32 (1.01‐1.72)	.041	0.734

We divided into quartiles by SBP variability after dividing into two groups by baPWV. We categorized the highest quartile (Q4) and the other quartile (Q1‐3). Adjusted factors for Model 1 included age, sex, body mass index, diabetes, total‐cholesterol, high‐density lipoprotein cholesterol, smoking, alcohol, pre‐existing cardiovascular disease, the use of antihypertensive drug and statin, office SBP, and average home SBP. Model 2 added log‐transformed NT‐proBNP to adjusted factors in model 1. ARV indicates average real variability; baPWV, brachial‐ankle pulse wave velocity; CI, confidence interval; CV, coefficient of variation; HR, hazard ratio; SBP, systolic blood pressure; SD, standard deviation. .

In mediation analysis, the proportions of the association between each index of BP variability and CVD events mediated by higher baPWV were −1.0%, −0.8, and −2.4% for SD_SBP_, CV_SBP_ and ARV_SBP_, respectively.

## 4 DISCUSSION

The results of our present analyses demonstrated that day‐by‐day home BP variability was associated with CVD events in the group with higher baPWV values, independently of traditional cardiovascular risk factors including office BP and average home BP in this practitioner‐based population with more than one cardiovascular risk, whereas this association was not found in the group with lower baPWV values.

To the best of our knowledge, this study is the first to show that arterial stiffness may be a moderating factor on the association between home BP variability and CVD events. Our results would be explained by a possible pathogenetic link proposed as the systemic hemodynamic atherothrombotic syndrome (SHATS) hypothesis.^27^, ^28^, ^29^ CVD risk factors (hypertension, dyslipidemia, diabetes, CKD, smoking, inflammation, etc.) cause endothelial dysfunction, which leads to the progression of atherosclerosis and arteriosclerosis. BP fluctuation (ie, BP variability) triggers atherosclerotic plaque rupture, which can result in coronary artery disease and atherothrombotic cerebral infarction. Accordingly, the SHATS hypothesis indicates that BP variability increases arterial stiffness and vice versa, leading to a vicious cycle of the association between BP variability and arterial stiffness; this association provides not only the risk but also the trigger for CVD events.

In the previous reports, increased day‐by‐day home BP variability was associated with CVD events. We previously reported that day‐by‐day home BP variability was associated with CVD events in the population of the J‐HOP study.^12^ Another study also demonstrated that home BP variability assessed by SD_SBP_ was associated with CVD events in 2455 patients without CVD risk from a general Japanese population.^11^ Concerning the association between baPWV and CVD incidence, Takashima and colleagues. reported that higher baPWV (≥1800 cm/s) was significantly associated with an increased CVD risk in the general Japanese population.^25^ In several guidelines, carotid‐femoral pulse wave velocity (c‐fPWV) has been recommended as an index of arterial stiffness.^30^ The value of c‐fPWV as an index of organ damage is more than > 10 m/s.^31^ According to previous studies, a baPWV value of ≥1800 cm/s (18 m/s) is equivalent to a c‐fPWV value of 10 m/s,^24^, ^25^, ^26^, ^32^, ^33^ which was associated with organ damage^31^ and all‐cause and CVD.^34^ Thus, the prognostic impact of increased BP variability and arterial stiffness for CVD incidence has been shown separately. Our findings thus provide the missing link between day‐by‐day home BP variability and arterial stiffness for CVD incidence. The findings of a previous study support our present findings. In a previous study about the association of BP variability assessed by ambulatory blood pressure monitoring and cardiovascular mortality, a higher ARV of 24‐h DBP was significantly predictive of cardiovascular mortality in untreated hypertensive but not normotensive patients.^35^ It has been considered that untreated hypertensives have greater progression of arterial stiffness compared with normotensive patients.

In the present study, the association between day‐by‐day home BP variability and CVD incidence remained after adjustment for NT‐proBNP. We previously reported that home BP variability was associated with the NT‐proBNP level in the patients with higher baPWV values, independently of traditional cardiovascular risk factors including office BP and average home BP, whereas this association was not found in the group with lower baPWV values in this cohort.^36^ Cardiac overload may be one of the phenotypes represented as the relationship between BP variability and arterial stiffness. The major determinant of serum NT‐proBNP level is left ventricular wall stress, especially during systole. The wall stress is determined by both left ventricular cavity pressure and radius. Arterial stiffness increases afterload and accelerates the effects of BP variability on afterload. The influence of BP variability on CVD increases in hard blood vessels, and one of the factors underlying this phenomenon may be the involvement of cardiac overload. In this study, day‐by‐day home BP variability, baPWV and NT‐proBNP were only measured at baseline. Further studies will be needed to determine whether increased NT‐proBNP as a result of the coincidence of higher day‐by‐day home BP variability and arterial stiffness is directly related to CVD incidence.

### 4.1 Limitations

This study has some limitations. First, because this was an observational study, we were unable to determine causality in the findings. Second, we assessed a practitioner‐based population with at least one cardiovascular risk factor; our findings may not be applicable to populations with lower cardiovascular risk or generalizable to other racial/ethnic and practical groups, and they must be verified in other groups. Third, higher home BP variability did not significantly increase the risk of CVD events in the lower baPWV group, which may have been due to the insufficient sample size, because of the nature of post‐hoc analysis.

## 5 CONCLUSIONS

Day‐by‐day home BP variability was associated with the CVD events in a group with higher baPWV values but not in a group with lower baPWV values in a practitioner‐based population with at least one cardiovascular risk factor.

## 6 PERSPECTIVES

Several hypertension guidelines^7^, ^8^, ^9^ indicate that the BP level and cardiovascular risk are appropriate targets of treatment and that BP variability predicts the prognosis of CVD, but the clinical implication of the vascular component for the management of hypertension has not been clear. Our present results demonstrate that the prognostic impact of home BP variability for CVD events differs according to the degree of arterial stiffness. Clinicians may therefore need to consider arterial stiffness in addition to BP variability when treating hypertension. However, there are no randomized control trial data to support this assertion. In the future, additional studies will be needed to determine whether the suppression of both arterial stiffness progression and BP variability provides a synergistic effect in reducing CVD events that exceeds the effects of targeting either arterial stiffness or BP variability alone.

## CONFLICT OF INTEREST

K. Kario received research funding from Omron Healthcare Co., Fukuda Denshi, and A&D Co.

## AUTHOR CONTRIBUTIONS

Kazuomi Kario takes primary responsibility for this paper.

Yusuke Ishiyama wrote the manuscript and did the statistical analysis.

Satoshi Hoshide and Hiroshi Kanegae helped with the statistical analysis.

Kazuomi Kario and Satoshi Hoshide collected the patients’ data.

Kazuomi Kario acquired research grants for the J‐HOP study.

Kazuomi Kario and Satoshi Hoshide reviewed/edited the manuscript.

## SOURCES OF FUNDING

This study was financially supported in part by a grant from the 21st Century Center of Excellence Project run by Japan's Ministry of Education, Culture, Sports, Science, and Technology (MEXT); a grant from the Foundation for the Development of the Community (Tochigi); a grant from Omron Healthcare Co., Ltd.; a Grant‐in‐Aid for Scientific Research (B; 21390247) from The Ministry of Education, Culture, Sports, Science, and Technology of Japan, 2009 to 2013; and funds from the MEXT‐supported program for the Strategic Research Foundation at Private Universities, 2011 to 2015 Cooperative Basic and Clinical Research on Circadian Medicine (S1101022) to K. Kario. The funding sponsors had no role in the study design or conduct of the study; in the collection, management, analysis, or interpretation of the data; in the preparation of the article; or in the decision to submit the article for publication.

## Supporting information

Supporting material
