# Supplementary material for: Impact of home blood pressure variability on cardiovascular outcome in patients with arterial stiffness: Results of the J‐HOP study
Source: J Clin Hypertens (Greenwich). 2021 Jul 20;23(8):1529–37. doi: 10.1111/jch.14327 (PMC8678810; doi:10.1111/jch.14327)
Supplement: Supplementary file 1 — Supporting material [file JCH-23-1529-s001.docx]

**SUPPLEMENTARY MATERIAL**

**Impact of home blood pressure variability on cardiovascular outcome in patients with arterial stiffness:**

**Results of the J-HOP Study**

Yusuke Ishiyama, MD^1^; Satoshi Hoshide, MD, PhD^1^; Hiroshi Kanegae^2^; and Kazuomi Kario, MD, PhD^1^

^1^Division of Cardiovascular Medicine, Department of Medicine, Jichi Medical University, Tochigi, Japan

^2^Genki Plaza Medical Center for Health Care, Tokyo, Japan

**Methods**

*J-HOP study*

The Japan Morning Surge-Home Blood Pressure (J-HOP) Study is a prospective observational study evaluating the use of home blood pressure (BP) to predict cardiovascular events in Japanese individuals with any of the following cardiovascular risk factors: hypertension, impaired glucose tolerance or diabetes mellitus, dyslipidemia, current smoking (and/or current chronic obstructive pulmonary disease), chronic kidney disease (CKD), atrial fibrillation, metabolic syndrome, and sleep apnea syndrome. The exclusion criteria for the J-HOP Study were a recent history of cardiovascular disease events (within 6 months), current hemodialysis treatment, chronic inflammatory disease, and malignancy. Diagnostic criteria of the cardiovascular risk factors were hypertension, defined as an office systolic BP (SBP) >140 mmHg and/or a diastolic BP (DBP) >90 mmHg, or current use of antihypertensive medication; impaired fasting glucose, defined as a fasting glucose level >110 mg/dl; impaired glucose tolerance, defined as a glucose level >140 mg/dl at 2 hr after a 75-g oral glucose tolerance test; diabetes, defined as a fasting glucose level >126 mg/dl and/or a casual glucose level >200 mg/dl or treated diabetes; hyperlipidemia, defined as a total cholesterol level >240 mg/dl or treated hyperlipidemia; CKD, defined as the presence of proteinuria or a value of <60 ml/min/1.73m^2^ for the estimated glomerular filtration rate^1^; metabolic syndrome, defined according to the guidelines of the Examination Committee of the Criteria for Metabolic Syndrome in Japan published in April 2005^2^; and sleep apnea syndrome, defined as an apnea-hypopnea index of >15 events/hr by overnight sleep polysomnography.

In Japan, there are 47 administrative divisions (prefectures). In 25 of the prefectures (Tochigi, Aichi, Yamaguchi, Nagano, Miyazaki, Ibaraki, Hiroshima, Kumamoto, Hyogo, Tottori, Chiba, Saitama, Niigata, Fukushima, Oosaka, Shiga, Gunma, Kanagawa, Tokyo, Toyama, Mie, Yamagata, Gifu, Saga, Nara), a total of 75 doctors at 71 institutions (45 primary practices, 22 hospital-based outpatient clinics, and 4 specialized university hospitals) agreed with the aims of this study and collected prospective data from individuals who agreed to participate in this project.

*Participants and participating centers*

Kazuomi Kario: Jichi Medical University School of Medicine; Satoshi Hoshide: Jichi Medical University School of Medicine; Hajime Haimoto: Haimoto Clinic; Kayo Yamagiwa: Yamagiwa Clinic; Kiyoshi Uchiba: Oooka Clinic; Syouichirou Nagasaka: Jichi Medical University School of Medicine; Yuichiro Yano: Nango Clinic; Kazuo Eguchi: Jichi Medical University School of Medicine and International University of Health and Welfare Hospital; Yoshio Matsui: Jichi Medical University and Hagi City Mishima Clinic; Motohiro Shimizu: Ogi City Fukukawa Clinic and Heigun Clinic; Akira Nakamura: Chukyo Clinic; Joji Ishikawa: Jichi Medical University School of Medicine and Koga Red Cross Hospital; Shizukiyo Ishikawa: Jichi Medical University School of Medicine and Washiya Hospital; Motoki Fukutomi: Simonoseki City Tsunoshima Clinic; Tomoyuki Kabutoya: Jichi Medical University School of Medicine and Ojikano Central Hospital and Chichibu Municipal Hospital; Kyousei Souda: Souda Clinic; Michiaki Nagai: Syoubara Red Cross Hospital and Syoubara City National Health Insurance Clinic; Seiichi Sibazaki: Ogi City Fukukawa Clinic; Hideyuki Uno: Jichi Medical University School of Medicine and Noda Hospital; Sachiyo Ogata: Joriku-Omiya Saiseikai Hospital; Yoshifumi Nojiri: Joetsu Community Medical Center Hospital; Ryuji Inoue: Kanzaki General Hospital; Kazuhiko Kotani: Tottori University Hospital; Satoshi Yamada: Yamada Clinic; Takeshi Mitsuhashi: Jichi Medical University School of Medicine; Hiroaki Tsukao: Yamashita Clinic; Tetsuya Aoki: Akasaki Clinic; Toshio Kuroda: Kuroda Internal Medicine and Cardiovascular Clinic; Yutaka Nakajima: Shimonoseki City Toyota Central Hospital; Akinori Hirai: Nagahama Red Cross Hospital; Hareaki Yamamoto: Yamamoto Clinic; Tsuneo Oowada: Oowada Internal Medicine and Gastrointestinal Clinic; Masaru Ichida: Jichi Medical University School of Medicine; Setsuko Katou: Katou Clinic of Internal Medicine; Takahiro Komori: Jichi Medical University School of Medicine and Utsunomiya Social Insurance Hospital and Kurai Kiyohiko Memorial Hospital; Sigeki Nishizawa: Nishizawa Clinic of Internal Medicine; Kazuhiro Murata: Ooshima Clinic; Takashi Utsu: Shiga Medical University; Toru Kato: Koyanagi Memorial Hospital; Osamu Kuwasaki: Kuwasaki Clinic of Internal Medicine; Yutaka Shimada: Kyaranoki Care Center; Yoshihiro Yonezawa: Yonezawa Clinic; Eiji Inoue: Inoue Clinic of Internal Medicine; Masatoshi Matsumoto: Jichi Medical University School of Medicine; Toru Kimura: Iiduka Clinic; Kenichi Sakakura: Kumano City Kiwa Clinic; Shingo Shikano: Ibuki Shikano Clinic; Kazuhiro Handa: Handa Clinic; Kouichirou Abe: Abe Clinic of Internal Medicine; Motoyuki Ishiguro: Ishiguro Clinic; Yoshio Onogaki: Onogaki Clinic; Hiroshi Kubo: Hiro Clinic of Cardiovascular Medicine and Gastrointestinal; Kouichi Tokai: Kamihira Clinic; Ryou Touji: Touji Clinic; Akiya Nakamoto: Nakamoto Clinic of Internal Medicine; Youichi Ehara: Yoshii Chuo Clinic; Masahiro Toshima: Kamiichi General Hospital; Nobuyuki Adachi: Adachi Clinic of Internal Medicine; Nobuo Takahashi: Takahashi Family Clinic; Masashi Tanaka: Manba Clinic; Fumihiko Eto: Privcare Family Clinic; Masahisa Shinpo: Jichi Medical University School of Medicine; Katsumi Tanaka: Youga Urban Clinic; Takeshi Takemi: Clinic Jingu-Mae; Masayuki Nagata: Nakata Clinic; Yukihiro Hojo: Jichi Medical University School of Medicine; Yoko Hoshide: Satou Clinic; Fumihiko Yasuma: Suzuka National Hospital; Hajime Yanagisawa: Sudou Hospital; Yukitaka Anraku: Omocyanomachi Internal Medicine Clinic; Shuichi Ueno: Jichi Medical University School of Medicine; Ryousuke Kusaba: Saitama Tsukuba Hospital; Naoshi Suzuki: Washiya Hospital; Nobuyuki Maki: Kamogawa City National Health Insurance Hospital.

(75 physicians and 71 institutes)

| Suppl. Table 1. Baseline clinical characteristics of included and not included subjects. | | | |
| --- | --- | --- | --- |
| **Descriptive variable** | **Not included (n=1583)** | **Included (n=2648)** | **P values** |
| Age, yrs | 64.9±10.2 | 64.9±11.4 | 0.942 |
| Men, % | 50.1 | 44.7 | 0.001 |
| Body mass index, kg/m^2^ | 24.0±3.4 | 24.4±3.5 | <0.001 |
| Current smoker, % | 13.6 | 11.4 | 0.033 |
| Daily drinker, % | 35.1 | 22.7 | <0.001 |
| Antihypertensive medication, % | 84.7 | 75.8 | <0.001 |
| Diabetes mellitus, % | 24.8 | 24.2 | 0.704 |
| Statin use, % | 22.3 | 24.4 | 0.127 |
| Pre-existing CVD, % | 10.6 | 13.9 | 0.002 |
| Total cholesterol, mg/dL | 206.8±32.9 | 199.7±32.5 | <0.001 |
| High-density lipoprotein, mg/dL | 59.0±16.1 | 56.7±14.7 | <0.001 |
| NT-proBNP, pg/mL | 46.7(23.1-88.1) | 53.8 (27.4-108.9) | <0.001 |
| **BP and PR parameters** |  |  |  |
| Office SBP, mmHg | 141.0±16.3 | 141.5±16.5 | 0.353 |
| Office DBP, mmHg | 81.1±10.1 | 81.3±10.9 | 0.580 |
| Office PR, bpm | 71.2±10.6 | 71.2±11.0 | 0.981 |
| Morning home SBP, mmHg | 136.0±15.4 | 139.8±15.9 | <0.001 |
| Morning home DBP, mmHg | 78.1±9.3 | 79.7±10.4 | <0.001 |
| Morning PR, bpm | 66.1±9.2 | 65.3±8.9 | 0.004 |
| Evening home SBP, mmHg | 129.2±14.4 | 130.6±15.2 | 0.003 |
| Evening home DBP, mmHg | 72.3±9.3 | 72.8±9.9 | 0.099 |
| Evening PR, bpm | 70.2±9.8 | 69.7±9.5 | 0.125 |
| Mean morning & evening SBP, mmHg | 132.6±13.8 | 135.2±14.5 | <0.001 |
| Mean morning & evening DBP, mmHg | 75.2±8.7 | 76.2±9.6 | <0.001 |
| Mean morning & evening PR, bpm | 68.2±9.0 | 67.5±8.7 | 0.019 |
| **BP variability parameters** |  |  |  |
| SD of morning SBP, mmHg | 8.4±3.4 | 8.3±3.3 | 0.357 |
| SD of evening SBP, mmHg | 9.6±3.7 | 9.7±3.8 | 0.304 |
| SD of morning & evening SBP, mmHg | 6.8±2.7 | 6.8±2.6 | 0.965 |
| Data are mean±SD, median (IQR), or percentage. baPWV indicates brachial-ankle pulse wave velocity; CVD, cardiovascular disease; DBP, diastolic blood pressure; IQR, interquartile range; NT-proBNP, N-terminal pro-B-type natriuretic peptide; SBP, systolic blood pressure; SD, standard deviation; PR, pulse rate. | | | |


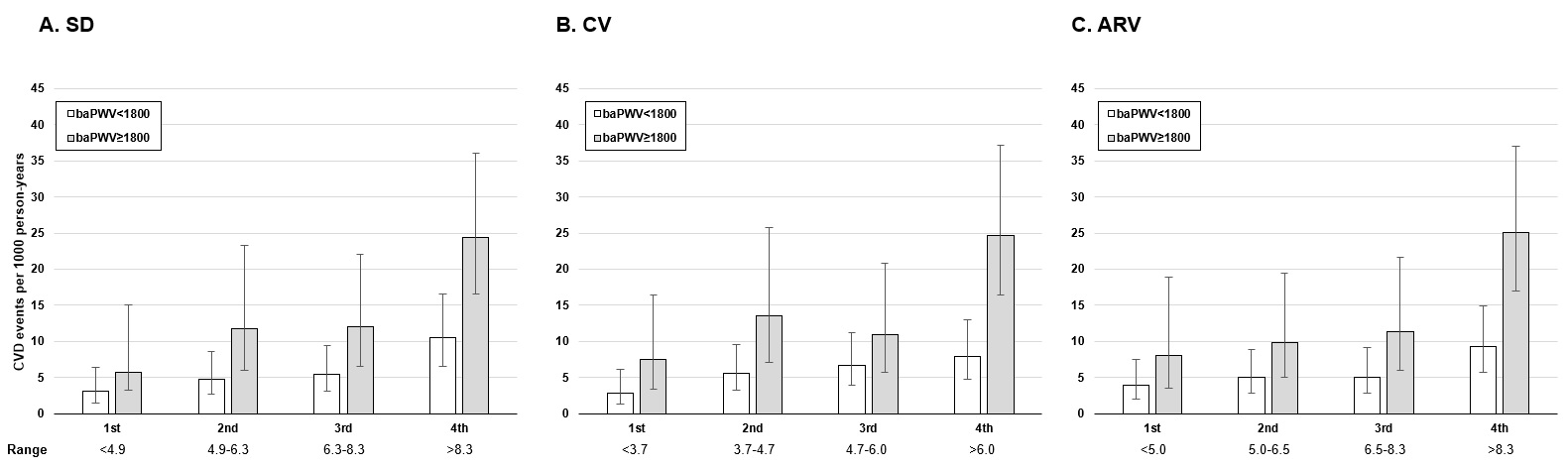


**Suppl. Figure 1.** CVD events per 1000 person-years of (A) SD (B) CV or (C) ARV of mean SBP. After dividing each day-by-day home SBP variability index into quartiles, we divided the group into lower (<1800 cm/s) and higher baPWV (≥1800 cm/s). Gray bars show the results for the higher baPWV group and white bars show those for the lower baPWV group. The error bars show the 95% confidence intervals.

| Suppl. Table 2. Hazard ratio of the quartile of BP variability in the population with lower or higher baPWV | | | | | | | | |
| --- | --- | --- | --- | --- | --- | --- | --- | --- |
| **PWV <1800 cm/s** | | | | | **PWV ≥1800 cm/s** | | | |
|  | **range** | **event/number** | **HR (95%CI)** | **P** | **range** | **event/number** | **HR (95%CI)** | **P** |
| SD of SBP, mmHg | | | | | | | | |
| Quartile 1 | <4.9 | 7/504 | 1 [Reference] |  | <4.9 | 4/154 | 1 [Reference] |  |
| Quartile 2 | 4.9-6.3 | 11/488 | 1.20 (0.46–3.14) | 0.712 | 4.9–6.3 | 8/166 | 2.06 (0.62–6.88) | 0.241 |
| Quartile 3 | 6.4-8.3 | 12/463 | 1.12 (0.43–2.93) | 0.815 | 6.3–8.3 | 10/189 | 2.41 (0.74–7.80) | 0.144 |
| Quartile 4 | >8.3 | 18/382 | 1.51 (0.58–3.96) | 0.399 | >8.3 | 25/280 | 4.52 (1.53–13.40) | 0.006 |
| CV of SBP, % | | | | | | | | |
| Quartile 1 | <3.7 | 6/474 | 1 [Reference] |  | <3.7 | 6/188 | 1 [Reference] |  |
| Quartile 2 | 3.7-4.7 | 13/499 | 1.53 (0.57–4.07) | 0.397 | 3.7-4.7 | 9/163 | 2.04 (0.72–5.81) | 0.183 |
| Quartile 3 | 4.7-6.0 | 14/453 | 1.56 (0.58–4.12) | 0.376 | 4.7-6.0 | 9/209 | 1.96 (0.69–5.57) | 0.210 |
| Quartile 4 | >6.0 | 15/411 | 1.59 (0.60–4.20) | 0.347 | >6.0 | 23/251 | 3.89 (1.56–9.73) | 0.004 |
| ARV of SBP, mmHg | | | | | | | | |
| Quartile 1 | <5.0 | 9/509 | 1 [Reference] |  | <5.0 | 5/153 | 1 [Reference] |  |
| Quartile 2 | 5.0-6.5 | 11/479 | 0.78 (0.32–1.93) | 0.595 | 5.0-6.5 | 8/185 | 1.07 (0.34–3.35) | 0.904 |
| Quartile 3 | 6.5-8.3 | 11/454 | 0.81 (0.33–2.00) | 0.642 | 6.5-8.3 | 9/209 | 1.45 (0.48–4.38) | 0.516 |
| Quartile 4 | >8.3 | 17/395 | 1.08 (0.45–2.60) | 0.866 | >8.3 | 25/264 | 2.78 (1.01–7.67) | 0.048 |
| We divided subjects into two groups by baPWV after dividing them into quartiles by SBP variability. Results were adjusted for age, sex, body mass index, diabetes, total-cholesterol, high-density lipoprotein cholesterol, smoking, alcohol, pre-existing cardiovascular disease, the use of antihypertensive drugs and statins, office SBP, and average home SBP. ARV indicates average real variability; baPWV, brachial-ankle pulse wave velocity; CI, confidence interval; CV, coefficient of variation; HR, hazard ratio; SBP, systolic blood pressure; SD, standard deviation. | | | | | | | | |

**References**

1. Matsuo S, Imai E, Horio M, et al. Revised equations for estimated GFR from serum creatinine in Japan. *Am J Kidney Dis* 2009;53:982-992.

2. Committee to Evaluate Diagnostic Standard for Metabolic Syndrome. Definition and the Diagnostic standard for metabolic syndrome. *Nippon Naika Gakkai Zasshi* 2005;94:794-809. (in Japanese).
